# Risk prediction and prevention in patients with advanced subclinical atherosclerosis

**DOI:** 10.1186/s12872-026-05968-6

**Published:** 2026-05-15

**Authors:** Michael J. Blaha, Abdulhalim J Kinsara, Abdulla Shehab, Afaf AlShammary, Eman Youssef, Ihab Attia, Islam Shawky, Loai Abudaqa, Magdy Abdelhamid, Mohamed I Lotfy, Salah Abusnana, Sameh Elkaffas, Saud Al Sifri, Waleed Al Habeeb, Amer M Johri

**Affiliations:** 1 Ciccarone Center for the Prevention of Cardiovascular Disease, Johns Hopkins School of Medicine, Baltimore, Maryland USA; 2 Cardiology, Ministry of National Guard-Health Affairs, King Saud Bin Abdulaziz University for Health Sciences, College of Medicine-Western Region, King Abdullah International Medical Research Center, Jeddah, Saudi Arabia; 3 College of Medicine and Health Sciences, United Arab Emirates University, Al Ain, United Arab Emirates; 4 Diabetes Center, Department of Medicine, King Abdulaziz Medical City, Riyadh, Kingdom of Saudi Arabia; 5 Diabetes, Lipidology & Metabolism Unit, Internal Medicine Department, Faculty of Medicine, Alexandria University, Alexandria, Egypt; 6 Department of Cardiology, Faculty of Medicine, Ain Shams University, Cairo, Egypt; 7 Department of Cardiology, Faculty of Medicine, Al Azhar University, Cairo, Egypt; 8 Al Qassimi Hospital, Sharjah, UAE; 9 Department of Cardiology, Faculty of Medicine, Cairo University, Giza, Egypt; 10 Department of Cardiology, Faculty of Medicine, Alexandria University, Alexandria, Egypt; 11 Diabetes and Endocrinology Department, University Hospital Sharjah, Sharjah, United Arab Emirates; 12 Al Hada Military Hospital, Taif, Saudi Arabia; 13 Department of Cardiac Sciences, Saudi Heart Association, King Saud University, Riyadh, Saudi Arabia; 14 Queen’s University, Kingston, Ontario Canada

## Abstract

**Background:**

Cardiovascular diseases (CVD) continue to be the leading cause of global mortality. Despite the alarming statistics, effective prevention of CVD remains a significant challenge in practice. The available risk stratification tools have critical limitations in the early detection of CVD. To address these gaps, it is crucial to integrate additional risk detection methods for more accurate identification of at-risk patients.

**Objectives:**

This article addresses the limitations of conventional CVD risk factors and emphasizes the need for individualized risk evaluation. Additionally, it evaluates the role of imaging techniques in the early detection of CVD and the personalized use of aspirin therapy when subclinical atherosclerosis becomes advanced.

**Methods:**

This article is based on an expert literature review and reflects the outcomes of a medical advisory board meeting that was held in the Middle East (ME) region. A multidisciplinary group of experts discussed the “cardiac risk continuum” concept and the importance of advanced subclinical atherosclerosis detection beyond traditional binary CVD classification. Experts evaluated the clinical feasibility of utilizing carotid ultrasound and coronary artery calcium (CAC) scoring, and assessed the role of aspirin in primary prevention for at-risk patients.

**Results:**

The need for tailored risk assessment strategies and individualized preventive measures was highlighted. The experts agreed on the practical use of CAC scoring and/or carotid ultrasound to identify at-risk patients and quantify subclinical atherosclerosis. Data suggest that aspirin estimated benefit increases proportionally with atherosclerosis burden and becomes a net-positive at CAC > 100 or carotid plaque score above 2.

**Conclusions:**

The experts emphasized the importance of individualized screening strategies tailored to the Middle Eastern population, considering the challenges and resource limitations in the region. They recommended selective use of CAC and carotid ultrasound to improve risk stratification and to guide a more personalized approach to managing CVD. The experts stressed the need for standardized protocols, healthcare providers’ education, and infrastructure development to ensure the effective implementation of these approaches.

## Introduction

Cardiovascular diseases (CVD), primarily ischemic heart disease (IHD) or coronary heart disease (CHD), and stroke, are the leading cause of death worldwide and play a significant role in causing disability [1–3]. In 2021, the global impact of CVD was substantial, affecting over 500 million individuals and resulting in approximately 20 million deaths [4, 5]. Specifically focusing on the Middle East (ME) region, the prevalence of CHD in 2021 was estimated at 10.1% [6]. Table 1 provides an overview of the burden of CVD, including incidence, prevalence, deaths, and disability-adjusted life years (DALY), for countries in the ME region in 2019 [7, 8].

**Table 1:** Estimates of the global burden of disease study on the incidence, prevalence, deaths, and DALY due to CHDs in some of the ME countries in 2019 [6, 7]

	CHD incidence	CHD prevalence	CHD deaths	DALY due to CHD
Bahrain	6,461 (5,475–7,566)	49,534 (45,552– 54,048)	863 (698–1,060)	23,147 (18,662– 28,406)
Egypt	450,548 (411,983–491,750)	3,263,746 (3,033,581–3,513,428)	181,885 (138,959– 233,632)	4,419,938 (3,313,613– 5,806,684)
Jordan	37,937 (32,682– 43,525)	310,388 (286,477– 336,917)	6,111 (5,196–7,286)	147,481 (124,436– 177,498)
Kuwait	16,837 (14,644– 19,359)	136,260 (125,999– 147,518)	2,599 (2,165–3,105)	71,677 (59,489– 86,115)
Qatar	6,440 (5,456–7,538)	46,196 (42,121– 50,305)	830 (631–1,068)	24,220 (18,355– 31,467)
Saudi Arabia	108,673 (96,041– 122,753)	835,249 (771,862– 903,746)	29,689 (24,089– 36,176)	883,559 (702,673– 1,099,540)
United Arab Emirates	27,614 (23,612– 32,368)	197,600 (180,763– 216,933)	4,880 (3,504–6,755)	174,392 (124,284– 243,500)

Despite these overwhelming statistics, deaths could be lowered by taking necessary precautions via primary and secondary prevention methods; however, preventing CVD remains a major challenge in clinical practice [9]. Generally, patients with CVD risk have been stratified into either primary or secondary prevention groups based on the absence or presence of a symptomatic CVD diagnosis [10].

Traditional CVD risk scores have some limitations in accurately addressing individualized risk. These scores may not encompass all relevant information contributing to an individual’s risk [11]. On the other hand, imaging tools such as coronary artery calcium (CAC) scoring and carotid ultrasound provide additional information that enhances risk assessment [12, 13]. These imaging techniques can identify individuals who may benefit from more aggressive treatment strategies or more personalized approaches that balance risk and benefit [14–17].

## Objectives

The main objective of this article is to document the key findings and outcomes of the medical advisory board meeting that took place in Egypt with a group of healthcare experts from different countries in the ME region. They aimed to discuss the limitations of conventional CVD risk factors and the necessity for individualized risk evaluation of patients, in addition to evaluating the concept of “Cardiac Risk Continuum”, that risk is not binary primary vs. secondary, but rather a continuous concept, with the growing recognition of an intermediate-advanced subclinical atherosclerosis stage.

The experts evaluated the existing body of evidence regarding imaging techniques and their importance in accurately identifying CVD. Additionally, they aimed to assess the clinical feasibility and value of carotid ultrasound imaging through a comprehensive review of current evidence and emerging research outcomes. Furthermore, they discussed the potential utilization of carotid ultrasound and CAC scoring, along with evaluating the potential benefits of aspirin in primary prevention for individuals at risk of developing CVD.

## Methodology

This article is the result of an expert analysis of current literature and the outcomes of a medical advisory board meeting held on 25 May 2023 in Egypt. The meeting brought together healthcare experts from various countries in the ME region, including Saudi Arabia, Egypt, the United Arab of Emirates, the United States (U.S.), and Canada. The review focused on the concept of a “Cardiac Risk Continuum” and emphasized the importance of detecting advanced subclinical atherosclerosis based on recent clinical data. Key search terms included “cardiovascular disease,” “risk factors,” “subclinical atherosclerosis,” “carotid ultrasound,” “coronary artery calcium,” and “aspirin therapy”. The databases used were PubMed, Scopus, and Web of Science, focusing on publications from the year 2022. Additionally, the feasibility of using aspirin therapy as a tool for lowering risk in the setting of advanced subclinical atherosclerosis detected via CAC or carotid ultrasound was discussed.

### Addressing the diagnostic gaps in the early detection of CVD

#### Challenging the current binary classification of primary vs. secondary prevention of CVD

The standard of care typically categorizes patients into two groups: those who have already experienced cardiovascular events and require secondary prevention and those who are at risk and should receive primary prevention [18]. This binary model approach acknowledges that CVD are multifactorial and result from a combination of various factors rather than a single cause [19]. Risk modifiers such as age, gender, family history, and comorbidities like diabetes play a crucial role in accurately assessing individual risk. However, by identifying actual subclinical diseases in patients, healthcare providers can prioritize the treatment approach that moves beyond the traditional risk factor model [18]. Recognizing that risk exists on a spectrum, physicians can adopt a personalized approach known as the risk continuum [20, 21].

When considering the current approach to CVD prevention, several challenges become apparent. One of these challenges stems from the limitations imposed by the binary model of primary versus secondary prevention [18]. This model fails to acknowledge the continuum of risk that individuals may fall into, thereby restricting the ability to tailor preventive strategies. Furthermore, the population-based approach to risk assessment hampers the personalization of risk prediction, as it may not consider an individual’s unique risk factors and characteristics [22].

During the meeting, experts highlighted the limitations of the binary model, which fails to consider the role of subclinical disease detection in reclassifying at-risk patients [20, 21]. By incorporating advanced risk assessment tools, healthcare professionals can achieve more precise risk stratification and provide tailored preventive measures. This may involve lifestyle modifications, close monitoring of risk factors, and targeted pharmacotherapy to effectively manage and reduce the risk of CVD on an individual level.

#### Addressing the gaps in the early detection of CVD using the currently available risk stratification tools

Risk prediction tools offer the advantage of estimating disease prognosis while providing impartial probabilities of various outcomes [23]. In theory, utilizing cardiovascular risk prediction models for the detection of CVD can yield significant benefits, such as early detection and reduction in the overall mortality rates [24]. However, there are diagnostic gaps that CVD patients often encounter [25, 26]. Cardiovascular disease assessment tools have many limitations, specifically their applicability to diverse populations. For example, the Framingham Risk Score (FHS) which incorporates smoking status, cholesterol levels, blood pressure, and the use of antihypertensive medication, as well as the Atherosclerotic Cardiovascular Disease (ASCVD) Risk Score for atherosclerotic cardiovascular disease. Both the FHS and the ASCVD Risk Score were derived from predominantly Caucasian cohorts, which limits their generalizability to other populations. This raises concerns about the accuracy of their extrapolation to other ethnic groups, including populations in ME [27, 28]. As a result, the assessment may not capture the full range of potential outcomes. They may also underestimate the risk in countries experiencing rising rates of CVD mortality and in diabetic patients, patients with central obesity, or patients with a family history of premature CVD. Additionally, these tools do not consider the socio-economic factors, which can significantly influence an individual’s risk profile [29].

Identifying at-risk patients before symptoms manifest is crucial to bridge these gaps and enable the early detection of CVD [22]. This can be achieved by implementing accessible screening tools that are available to all healthcare providers, establishing long-term management and treatment protocols for underlying risk factors, emphasizing prevention rather than merely treating symptoms, and adopting guidelines and recommendations that target subclinical at-risk groups [30].

Risk stratification has proven to be pivotal in various aspects of CVD, including identifying new indicators, assessing at-risk groups for targeted treatment, and improving the cost-effective utilization of therapy [31, 32]. Nowadays, the Pooled Cohort Equation is commonly employed, considering factors such as diabetes, smoking history, and basic health metrics, including blood pressure [33].

In summary, in the larger context of ASCVD prevention, an important debate is occurring as to whether we should continue risk factor screening or switch to disease screening by targeting early subclinical disease detection. For example, there is compelling observational data to suggest that risk classification is better with screening for CAC [13, 34].

- Expert Opinion.

The experts emphasized that the traditional binary model may not adequately address the complex nature of CVD. This model overlooks the impact of subclinical disease, which is important to accurately classify the at-risk patients. It fails to consider the risk continuum that a patient may fall into and hinders personalized risk prediction. To overcome these challenges, the experts suggested utilizing imaging tools like computed tomography (CT) scans and carotid ultrasound to enhance risk stratification and support the development of a more personalized management approach based on the extent of atherosclerosis detected.

They proposed that incorporating advanced imaging techniques, other risk factors, or biomarkers could further enhance risk prediction in primary prevention. Furthermore, they emphasized the importance of considering patients with advanced sub-clinical atherosclerosis who may require distinct treatment approaches.

### The potential impacts of identifying patents with advanced subclinical atherosclerosis

#### The cardiac risk continuum and advanced subclinical atherosclerosis

There is a growing need for a new perspective that considers atherosclerosis and ASCVD risk as a continuum [21, 35]. This approach would enable clinicians to more accurately align risk levels with the intensity of preventive therapy recommended [21]. Recent advancements in coronary imaging, specifically CAC scoring, have shed light on this matter. CAC serves as a reliable marker of atherosclerosis and predicts the risk of ASCVD in susceptible individuals [36].

A high CAC score indicates the accumulation of atherosclerosis over a patient’s lifetime, reflecting the burden of the disease and predicting future cardiovascular events. Fortunately, cardiac CT scans, which are widely available and dependable tools, could effectively assess the presence of subclinical atherosclerosis [37, 38].

Building upon these advancements, the 2018 ACC/AHA cholesterol guidelines reinforced the recommendation of considering CAC scores for patients in the intermediate risk category [39]. CAC scoring has the potential to reclassify patients with low to moderate cardiovascular risk, allowing for targeted preventive therapies such as statins and aspirin [38, 40]. Patients with high CAC scores are more likely to benefit substantially from these preventive interventions, while those with lower scores may experience limited advantages [20].

#### What are the clinical implications for patients identified as having ‘advanced subclinical atherosclerosis’ under this new paradigm?

Incorporating the concept of advanced subclinical atherosclerosis into risk-based management strategies can enhance risk assessment, tailor treatment approaches, provide effective patient education, and monitor disease progression more effectively. This comprehensive approach can potentially improve patient outcomes and reduce the burden of CVD [41].

Atherosclerosis is highly prevalent in the ME region. Patients in this region are often affected at a younger age compared to their European counterparts [7] Many patients in the ME region present with advanced calcification in their arteries, which poses challenges in disease management [42]. Additionally, screening guidelines for CVD are often based on European and American populations, which may not be entirely appropriate for Middle Eastern populations [7].

Convincing patients to undergo screening tests and adhere to medication regimens for CVD, especially when they do not exhibit symptoms, can be challenging [43]. During the board meeting, healthcare experts highlighted a lack of alignment between cardiologists and primary care physicians in implementing guidelines and screening tests for patients at risk of cardiovascular events in the ME region. Furthermore, limited resources in certain areas can undermine the effectiveness of relying solely on underlying risk factors for predicting cardiovascular events.

- Expert Opinion.

The experts emphasized the importance of performing individualized screening in the ME to identify high-risk patients at earlier stages. They advocated for tailoring screening guidelines specifically for the Middle Eastern populations and proposed using CAC scoring as a practical approach to identify at-risk individuals. Despite limited resources, the implementation of screening programs was deemed highly beneficial for early detection and prevention of CVD, particularly for individuals under the age of 40–50 who might not exhibit symptoms. However, the experts also emphasized the need for careful consideration of cost, benefits, and available resources when selecting screening tests. Cost-effective screening tests that can modify risk factors within the resource constraints of the region should be prioritized.

Given the influence of risk factors, such as hypertension, obesity, and genetic variations, the experts stressed the necessity of customizing guidelines specifically tailored for the Middle Eastern population. They highlighted the importance of verifying patient information and incorporating patient feedback to improve the efficiency of clinical practice. By considering these factors, healthcare providers can optimize the effectiveness of screening programs and ultimately improve overall cardiac care to the population at CVD risk in the ME.

### The feasibility and potential limitations of the clinical application of cardiac CT

#### Implementing cardiac CT in clinical settings

Cardiac CT scans play a crucial role in evaluating patient risk by detecting calcification and atherosclerosis [44, 45]. One simple tool derived from a non-contrast cardiac CT scan is the CAC score, which serves as a predictor for cardiovascular risk [46, 47]. A CAC score above 100 is considered to be at least moderate risk and is strongly associated with increased mortality rates compared to a CAC score of zero [48].

The specific range for a high CAC score can vary, typically falling above 100 to 400, depending on the age group. A recent study conducted on an asymptomatic primary prevention population demonstrated that the combination of CAC with the Pooled Cohort Equations led to a notable enhancement in risk discrimination for future CVD mortality. The integration of CAC improved the concordance or C statistics from 0.71 to 0.75 in middle-aged adults [49, 50]. Numerous studies have examined the relationship between CAC scores and mortality rates [49, 51, 52]. Their findings suggest that CAC scores around 400–1000 are associated with higher annual mortality rates comparable to those observed in patients who participated in secondary prevention trials such as the FOURIER trial [53].

The European Society of Cardiology (ESC) has recognized CAC scoring as the best-established imaging modality to improve CVD risk stratification. CAC scoring may be considered to improve risk classification around treatment decision thresholds (IIb). If CAC is detected, its extent should be compared with what would be expected for a patient of the same sex and age. Higher-than-expected CAC increases the person’s calculated risk, whereas absent or lower-than-expected CAC is associated with lower-than-calculated risk [54]. Moreover, findings indicate that patients with CAC scores ranging from 250 to 300 face risk comparable to those enrolled in the SPRINT trial and should receive aggressive blood pressure management [55, 56]. In 2020, the multi-ethnic study of atherosclerosis (MESA) suggested that patients with high CAC scores > 100 are projected to more likely benefit from aspirin therapy. As such, CAC appears to be superior to the pooled cohort equations alone in informing aspirin allocation in “primary prevention” [57].

### Cardiac CT for precision primary prevention “A place for high-intensity statins and aspirin”

Elevated CAC score is linked to a higher risk of ASCVD but no adjusted increase risk of bleeding. Individuals with a CAC score of ≥ 100 have mildly elevated bleeding risk yet five times greater ASCVD risk compared to those with a CAC score of 0. A high CAC score thus may help identify individuals who are likely to benefit from aspirin therapy in primary prevention of CVD, conditioned that they have a lower risk of bleeding and estimated ASCVD risk that is not low [58].

In 2022, the American College of Cardiology (ACC) released an expert consensus decision pathway on the use of non-statin medications, with a focus on low-density lipoprotein (LDL) goals. According to these guidelines, CAC scores should be considered for intermediate-risk patients. With scores above 100 or surpassing the 75th percentile, patients with consistently elevated LDL levels above 70 should receive moderate to high-intensity statin therapy and ezetimibe if needed. If their CAC score exceeds 1000, they should also be considered for treatment with a PCSK9 inhibitor and/or ezetimibe [59].

- Expert Opinion.

The experts highlighted that CAC scoring is a valuable tool for assessing CVD risk. However, it is important to exercise caution when interpreting the absence of CAC in certain populations, such as younger adults and women, to avoid delaying primary preventive interventions [60]. While CAC measurement is increasingly utilized, concerns have been raised regarding radiation exposure and healthcare costs associated with this method. The ACC/AHA guidelines have considered these factors in their recommendations [61, 62]. As an alternative to CAC scoring, carotid ultrasound can be used for plaque detection when CAC scoring is not feasible or available as recommended in ESC 2021 guidelines [12, 16, 54].

Moreover, the experts clarified that coronary computed tomography angiography (CCTA) is an essential noninvasive tool for evaluating coronary artery disease (CAD) and has been explored for assessing cardiovascular risk. Recent updates from the 2023 ESC and ACC/AHA guidelines now recognize its role in certain patients suspected of having CAD [62–64]. They added that aortic imaging has become increasingly important in evaluating aortic conditions that can impact cardiac risk. Numerous studies have demonstrated that both CCTA and CAC are reliable for risk assessment, yielding outcomes comparable to traditional imaging methods [65–67]. 

Despite the need for more data to establish the correlation between carotid and coronaries, carotid atherosclerosis remains a valuable indicator for assessing cardiovascular risk and guiding preventive strategies in clinical practice.

The experts also agreed on the wide adoption of CAC scoring in clinical practice. However, they recognized the need for expertise and implementation processes. They also noted a limitation of CAC scoring, highlighting that a high score within the range of 100 to 300 may be considered less abnormal in the older population aged > 75. Additionally, accessibility challenges for such tools were acknowledged, particularly in remote and indigenous communities.

### The impact of carotid ultrasound in identifying CVD risk

Screening for CVD, which includes atherosclerosis, is a critical priority for healthcare systems due to the substantial health expenditure associated with these conditions [30]. It has been projected that nearly half of the U.S. population would develop some form of CVD by 2030, with an estimation to reach close to 1 trillion dollars by that time [68].

To tackle these challenges, carotid ultrasound has been proposed as a cost-effective alternative for risk prediction and screening of CVD. This diagnostic method has several advantages, such as being radiation-free and less expensive compared to other tests. Moreover, it has proven useful in both screening and risk stratification, providing valuable information on long-term cardiovascular risk [69, 70].

Risk stratification involves utilizing plaque grades obtained from carotid ultrasound to categorize patients into low, intermediate, and high-risk groups [13]. This categorization is based on measuring plaque height and grade as well as lesion-modifying parameters like calcification and vulnerability. On the other hand, screening involves using carotid ultrasound as an affordable and user-friendly tool for screening asymptomatic individuals and larger populations [71–74].

- Expert Opinion.

During the meeting, the experts emphasized the significant challenges faced by physicians locally when it comes to implementing and scaling up CAC scoring and carotid ultrasound approaches. These challenges were primarily attributed to the lack of adequate training courses, the absence of standardizations, and poor infrastructure. While private practices have shown promising results, this approach cannot be easily extended to the entire population. The experts emphasized that the main barrier lies in the lack of implementation rather than the methods’ limitations.

To address these challenges, the experts discussed potential strategies to enhance the implementation and the impact of CAC scoring and carotid ultrasound. They proposed providing advanced training courses for physicians and technicians to ensure a competent workforce capable of effectively performing the tests. Additionally, they highlighted the importance of developing standardized protocols and procedures for CAC scoring and carotid ultrasound. Standardization would improve the consistency and reliability of the results across different healthcare settings.

The experts further suggested that adopting an affordable POCUS device could make carotid ultrasound more feasible, especially in settings where access to sophisticated imaging equipment might be challenging. As an application to their suggestions, a previous study demonstrated that a handheld ultrasound device could accurately measure carotid plaque parameters with good correlation and reliability compared to conventional ultrasound systems [75, 76]. The experts recommended starting with the implementation of these approaches in medium-risk groups before gradually expanding to cover the entire population. This phased approach allows for a gradual buildup of the necessary infrastructure, including the integration of additional ultrasound devices [68]. By implementing these recommendations, healthcare systems can overcome the existing challenges and improve the availability and accessibility of imaging modalities.

### Current and future access of POCUS in evaluating CVD

Point-of-care ultrasound (POCUS) has brought about a paradigm shift in diagnostic imaging, transforming the way clinicians approach patient care [77, 78]. Traditionally, patients were referred to radiologists for ultrasound examinations, which could be time-consuming and may not always yield immediate results. POCUS addresses this issue by placing the ultrasound device in the hands of the clinician at the patient’s bedside [79]. This eliminates the need to send patients to radiology departments and allows clinicians to perform focused ultrasounds using portable devices without the requirement of dedicated sites [80]. Consequently, POCUS offers real-time results, saves time, and enables prompt action on critical findings [81, 82].

Although POCUS has gained prominence in various medical specialties, the adoption of its use for assessing cardiovascular risk has been relatively slower among cardiologists [83, 84]. However, studies have demonstrated that POCUS can effectively detect carotid plaque and assess cardiovascular risk, making it a valuable tool in this context [85, 86]. The main challenge lies in educating and training clinicians to utilize POCUS accurately and interpret the results reliably [87].

The benefits of POCUS in cardiovascular risk assessment extend beyond healthcare settings. The ability to detect plaque by an ultrasound scan can serve as a motivating factor for patients to make necessary lifestyle changes to improve their cardiovascular health [86]. Handled POCUS devices have demonstrated comparable performance to full-sized ultrasound devices, making them a practical option for point-of-care use [78, 80]. To ensure widespread adoption, it is crucial to incorporate POCUS training into medical education programs, equipping future healthcare practitioners with the necessary skills [88]. The future of POCUS holds promising developments, including augmented reality tools for enhanced imaging and teaching, broader adoption across various medical specialties, and integration of ultrasound data with other healthcare metrics [88, 89].

However, barriers to accessing remote regions pose a significant challenge when it comes to providing POCUS education in those areas [90, 91]. To address this, tele-mentoring, a tool for remotely teaching and guiding providers, can be employed to facilitate the dissemination of POCUS learning to remote locations. With tele-mentoring, providers can learn by using their own equipment and apply what they learn to their patients, effectively retaining knowledge within their communities [92].

- Expert Opinion.

The experts have emphasized the need for customized strategies to tackle the challenges encountered in the ME region effectively. They have put forth a series of recommendations to comprehensively address these challenges. First, healthcare settings could obtain certifications from reputable institutions or organizations specializing in the field to ensure correct data interpretation and analysis application. Secondly, organizations could provide concise, targeted training courses that focus on teaching healthcare professionals to effectively interpret results and determine the appropriate next steps. In addition, training should be centralized in order to ensure that individuals are properly qualified and have met the necessary requirements to access training.

To enhance accessibility, the experts suggested utilizing POCUS, integrating carotid ultrasound into primary care, and providing training to healthcare staff for effective utilization and implementation of these approaches. Additionally, they emphasized the importance of considering the cost-effectiveness of screening and interventions when making decisions.

The experts unanimously agreed that individualized screening is crucial and that additional risk stratification can guide personalized treatment intensity. They advocated for considering all these factors to enable a tailored and effective approach to managing CVD.

### The value of carotid artery plaque score in shifting guidelines to aspirin therapy

Carotid plaque can serve as an indicator of atherosclerosis as it reflects the overall burden of atherosclerosis in the coronary arteries [17]. The presence of carotid atherosclerosis in individuals with low Framingham risk scores raises the potential for the necessity of screening subclinical atherosclerosis. Consequently, the quantification of carotid arterial plaque has become a significant tool for CVD risk stratification [27].

The carotid plaque score has demonstrated a strong predictive ability for major adverse cardiovascular events (MACEs) and may outperform the SCORE2 system in predicting CVD risk [93–95]. The carotid plaque score is a measure of the number of carotid segments afflicted by plaque on the left and right side of the body. A cutoff score of greater than 2 (at least carotid segments with plaque) appears to be effective in identifying individuals at high risk. Various measurements of carotid atherosclerosis have shown an association with overall atherosclerosis and CVD, indicating their unfulfilled potential in risk stratification. However, to enhance the clinical utility of the carotid plaque score, establishing a prognostic cutoff value is recommended to further improve its applicability in clinical practice [96].

Emerging research, including 5 different studies derived from the Multi-Ethnic Study of Atherosclerosis (MESA) [49], and the Atherosclerosis Risk in Communities (ARIC) study, has demonstrated that patients with moderate or greater carotid artery plaque might experience benefits from aspirin therapy [97]. However, researchers found that patients with only one small plaque are not expected to significantly benefit from aspirin treatment. Researchers finally concluded in their study that the benefit from aspirin therapy increases proportionally with increasing plaque score [49].

- Expert Opinion.

The meeting experts agreed that, in primary prevention, patients with a plaque score of 2 or higher appear to benefit most from aspirin therapy. They added that the efficacy of POCUS in detecting plaque and using aspirin in primary prevention needed to be definitively determined through randomized control trials (RCT). Nevertheless, the carotid plaque score appears suitable and reasonable for cardiovascular risk estimation, either as a lone risk assessment tool or in addition to cardiovascular risk prediction algorithms, and may guide decision-making for cardiovascular preventive therapies. Experts emphasized on the need for future studies to reinforce the evidence beyond carotid plaque score.

The experts recommended developing clear guidelines for assessing plaque scores and identifying high-risk patients with high plaque burden. They stressed the importance of providing adequate training and education to healthcare teams on using imaging modalities for risk stratification and that they should consider potential risks and benefits before initiating aspirin. They finally added that it was important to conduct trials to evaluate the impacts of plaque quantification on clinical decision-making to ensure optimum patient care.

### Creating a roadmap for the prediction and management of cardiovascular risk

In order to curb CVD effectively, it is important to adopt a comprehensive approach by considering the entire cardiac continuum and tailoring personalized treatments accordingly [63, 98]. It is imperative to prioritize early detection and interventions of atherosclerosis, as these are crucial strategies for effective disease management [63]. To achieve this, guidelines and action plans must be generated targeting individuals who are identified to benefit from additional preventive measures. Moreover, consensus among different healthcare disciplines needs to be reached on the appropriate utilization of the available prognostic tools. Consistent terminology should be employed to minimize confusion and encourage widespread acceptance [62–64]. Educating healthcare professionals is essential to raise awareness of the early detection of atherosclerosis through promising imaging modalities [99, 100].

Specialized cardiologists and primary care physicians could play crucial roles in screening for atherosclerosis in at-risk patients by utilizing carotid ultrasound and cardiac CT. Establishing a risk-benefit ratio is essential to justify appropriate management decisions for asymptomatic patients [23, 30].

- Expert Opinion.

The experts agreed that collaborative efforts are needed to standardize imaging biomarkers that could predict CVD risk. Consequently, protocols and recommendations from medical societies could establish CAC scoring and carotid ultrasound as potential standards in practice.

To enhance early detection and prevention, the experts recommended implementing educational campaigns on social media, sponsoring governmental initiatives, and collaborating with health authorities to improve access to imaging tools for the early identification of high-risk patients.

Finally, progress is needed in previously mentioned areas, such as guidelines, clinical trials, education, and protocols, to shift from population-based screening tools to personalized risk assessment tools.

## Conclusion

Tailored and individualized screening strategies are imperative in the ME region, considering the specific challenges and resource limitations while also recognizing the potential benefits of early interventions in preventing CVD. It may be necessary to adapt guidelines, tools, and approaches from other regions to suit the unique population and healthcare ecosystem of the ME. Moreover, a crucial shift needs to occur from conventional risk assessment to non-invasive imaging techniques that can accurately quantify CAC and plaque burden. This shift would enable the implementation of individualized risk assessment strategies. By utilizing these advanced imaging techniques, healthcare professionals can more precisely assess the extent of atherosclerosis, leading to an informed clinical decision on aspirin therapy.

By incorporating these recommendations, healthcare professionals in the ME region can significantly improve their ability to detect and manage CVD at earlier stages. This, in turn, will enhance patient outcomes and overall cardiovascular care in the region.

## Data Availability

Not applicable.
